# Effect of Xanthohumol, a Bioactive Natural Compound from Hops, on Adenosine Pathway in Rat C6 Glioma and Human SH-SY5Y Neuroblastoma Cell Lines

**DOI:** 10.3390/nu16111792

**Published:** 2024-06-06

**Authors:** Adrián Tejero, David Agustín León-Navarro, Mairena Martín

**Affiliations:** Department of Inorganic and Organic Chemistry and Biochemistry, Faculty of Chemical Sciences and Technologies, Institute of Biomedicine, IDISCAM, University of Castilla-La Mancha, Avenida Camilo José Cela 10, 13071 Ciudad Real, Spain; adrian.tejero@uclm.es (A.T.); mairena.martin@uclm.es (M.M.)

**Keywords:** xanthohumol, adenosine receptors, cell cultures

## Abstract

Xanthohumol (Xn) is an antioxidant flavonoid mainly extracted from hops (*Humulus lupulus*), one of the main ingredients of beer. As with other bioactive compounds, their therapeutic potential against different diseases has been tested, one of which is Alzheimer’s disease (AD). Adenosine is a neuromodulatory nucleoside that acts through four different G protein-coupled receptors: A_1_ and A_3_, which inhibit the adenylyl cyclases (AC) pathway, and A_2A_ and A_2B,_ which stimulate this activity, causing either a decrease or an increase, respectively, in the release of excitatory neurotransmitters such as glutamate. This adenosinergic pathway, which is altered in AD, could be involved in the excitotoxicity process. Therefore, the aim of this work is to describe the effect of Xn on the adenosinergic pathway using cell lines. For this purpose, two different cellular models, rat glioma C6 and human neuroblastoma SH-SY5Y, were exposed to a non-cytotoxic 10 µM Xn concentration. Adenosine A_1_ and A_2A_, receptor levels, and activities related to the adenosine pathway, such as adenylate cyclase, protein kinase A, and 5′-nucleotidase, were analyzed. The adenosine A_1_ receptor was significantly increased after Xn exposure, while no changes in A_2A_ receptor membrane levels or AC activity were reported. Regarding 5′-nucleotidases, modulation of their activity by Xn was noted since CD73, the extracellular membrane attached to 5′-nucleotidase, was significantly decreased in the C6 cell line. In conclusion, here we describe a novel pathway in which the bioactive flavonoid Xn could have potentially beneficial effects on AD as it increases membrane A1 receptors while modulating enzymes related to the adenosine pathway in cell cultures.

## 1. Introduction

Numerous natural bioactive molecules, such as polyphenols, flavonoids, alkaloids, and terpenes, have been discovered and studied as potential therapeutic candidates to act against neurodegenerative diseases [1]. One such compound belonging to the group of flavonoids is xanthohumol (Xn, 2′,4′,4-trihydroxy-6′-methoxy-3′-prenylchalcone), found in the hop flower (*Humulus lupulus*), one of the main ingredients of beer, which contributes at least 30% of its polyphenol content [2]. This prenylated chalcone has been reported to have anti-inflammatory [3,4], antioxidant [5,6,7], and antiproliferative properties [8] on various cell lines and animal models. It has also been postulated as a potential therapeutic agent against the neurodegeneration characteristic of Alzheimer’s disease (AD), where it reduces the accumulation of amyloid β-peptide and the hyperphosphorylation of the tau protein in cellular models of AD [9].

Neurodegenerative diseases comprise a type of neurological abnormality in which progressive neuronal population loss occurs in the central nervous system (CNS). This loss leads to several alterations in functions such as movement, learning, and memory. AD is the most predominant neurodegenerative disease worldwide [10]. The pathogenesis, however, has not yet been discovered, but several studies suggest that oxidative stress [11], mitochondrial dysfunction [12], excitotoxicity [13], and neuroinflammation [14] may be involved.

Excitotoxicity is a complex process that leads to neuronal loss and is triggered by overactivation of glutamate receptors due to an increased release of excitatory transmitters such as L-glutamate and the consequent intracellular calcium enhancement. Adenosine is a very well-known neuromodulator in the nervous system, where it can modulate the release of several neurotransmitters, including L-glutamate. Adenosine acts through four different types of receptors: A_1_, A_2A_, A_2B_, and A_3_. The activation of A_1_ and A_3_ receptors inhibits adenylate cyclase activity and reduces the level of glutamate released into the extracellular medium, whereas the activation of A_2A_ and A_2B_ causes an increase in extracellular glutamate levels [15].

Several studies carried out using human samples have reported that in AD, the A_1_ receptor is decreased in some brain regions such as the hippocampus [16], temporal cortex, and thalamus [17], while the A_2A_ receptor is increased in studies performed in animal models of AD [18,19,20,21] and in AD patients [22]. However, this increase does not translate into hyperexcitability in AD-affected regions, where A_2A_R preserves basal transmission [19,23]. Additionally, a previous study from our group has shown that the adenosinergic network is disrupted in the brains of human post-mortem AD patients [24].

Moreover, the adenosine network is involved in several pathological processes. The extracellular ATP release can act as a stress signal. Ecto-5′-nucleotidase, or CD73, converts AMP derived from this ATP into adenosine, which activates mainly A_2A_R, causing an overactivation of this receptor [25]. The enzymes converting adenosine into AMP, adenosine kinases [26], and equilibrative nucleoside transporters (ENT) have also been studied in the search for potential inhibitors of this network, as they can act as therapeutic agents against neurological diseases [27].

The search for possible natural bioactive compounds that modulate the functionality of adenosine receptors could, therefore, be very helpful in the prevention and identification of new therapeutic molecules for AD. In that sense, previous work carried out in our group has shown that Xn causes a modulation at the level of gene expression of adenosine receptors in SH-SY5Y cells [28]. However, until now, it has not been fully reported how it could affect the adenosinergic pathway, including levels of these receptors in the membrane, functionality, or related enzymatic activities. Due to the importance of this pathway in neurodegenerative diseases, the present study aims to characterize the effect of Xn on the adenosinergic pathway in cellular models of rat C6 glioma and human SH-SY5Y neuroblastoma.

## 2. Materials and Methods

### 2.1. Cell Culture

C6 rat glioma and human SH-SY5Y neuroblastoma cells were purchased from the American Type Culture Collection (Manassas, VA, USA) and were grown in a humidified atmosphere consisting of 95% air, 5% CO_2_, and at a temperature of 37 °C. For C6 cell culture medium, Dulbecco’s modified Eagle’s medium (DMEM) supplemented with 1% nonessential amino acids, 2 mM L-glutamine, 1% antibiotic–antimycotic, 10% decomplemented fetal bovine serum (FBS) (PAA, Pasching, Austria), and 50 µg/µL gentamicin (Gibco, Waltham, MA, USA) was used. For the SH-SY5Y human cell line medium, DMEM supplemented with 10% FBS and 1% antibiotic–antimycotic was used. At confluence, cells were detached from 10 mL Petri dishes (Nunc, Roskilde, Denmark) by mechanical action in the case of C6 or trypsin (TrypLE Express, Gibco, USA) in the case of SH-SY5Y cells. The detached cells were then transferred to other dishes as required depending on the experiment carried out, which included using 10 mL dishes for membrane isolation or 96-well dishes (Nunc, Roskilde, Denmark) for viability assays.

### 2.2. Cell Viability Assay

For cell viability measurement, an in vitro assay kit (Cell Proliferation Kit II, XTT, Roche, Manheim, Germany) based on the reduction of tetrazolium salt (XTT) to formazan, delivering a colorimetric signal, was used. C6 and SH-SY5Y cells were detached and counted on a TC 20™ Automated Cell Counter (Bio-Rad, Madrid, Spain) and seeded in 96-well dishes (10^4^ cells/well) for their exposure to xanthohumol (Sigma-Aldrich, Madrid, Spain) prepared following manufacturer indications at different concentrations during 24 h of treatment. Xn was solubilized in DMSO and then diluted in the corresponding cell medium, depending on the cell line. Then, the cells were incubated for 2 h with the XTT solution at 37 °C. The orange formazan color produced by viable cells was studied by recording the absorbance at 475 nm and 690 nm. Each single experiment was run in sextuplicate. Photographs were made with a digital camera (Leica DFC350FX) attached to a Leica DMI6000B (Leica Microsystems, Wetzlar, Germany) microscope.

### 2.3. RNA Isolation and cDNA Preparation

RNA isolation was performed using the PureLinkTM RNA Mini Kit from Invitrogen by Thermo Fisher Scientific (Waltham, MA, USA) (cat. number 12183018A) according to the manufacturer’s protocol. RNA purity was confirmed by measuring absorbance at 260 nm and 280 nm (the A260/A280 ratio) and making sure it was between 1.8 and 2.0. After that, retrotranscription of RNA was performed with a High-Capacity cDNA Archive Kit from Applied Biosystems (Waltham, MA, USA). RNA was maintained for 10 min at 25 °C and then incubated for 2 h at 37 °C before interrupting retrotranscription at 85 °C for 5 s.

### 2.4. Quantitative Real-Time RT-PCR Analysis

Gene expression was quantified by quantitative real-time RT-PCR in an ABI Prism 7500 Fast SDS, using 20 ng of cDNA for each reaction and TaqMan Universal PCR Master Mix following the manufacturer’s indications (Applied Biosystems, Foster City, CA, USA). The TaqMan probes used for experiments were for A_1_ (Hs00181231_m1), for A_2A_ (Hs00169123_m1, Rn00583935_m1), for AC1 (Adenylate cyclase 1) (Rn02115682_s1), and for β-actin (Hs99999903_m1, Rn00667869_m1). The reaction was conducted at 95 °C for 20 s, then followed by 40 cycles at 95 °C for 3 s and 60 °C for another 30 s. Gene expression was analyzed using the 7500 Fast System SDS 1.3.1 program. Each gene expression level was normalized to its endogenous control and relativized according to a calibrator using the following equation: RQ = 2 −ΔΔt = 2 − ((Ct target gene − Ct β-actin) sample − (Ct target gene − Ct β-actin) calibrator). All cDNA samples were run in duplicate, and the average represents a single value for each mRNA and sample.

### 2.5. Plasma Membrane Isolation Procedure

For cellular plasma membrane isolation, cells were detached from plates with a ThermoScientificTM NuncTM cell scraper. The cells were then resuspended and homogenized in an isolation buffer consisting of 50 mM Tris-HCl, pH 7.4, 10 mM MgCl_2_, and protease inhibitors, specifically phenylmethylsulfonyl fluoride and bacitracin at 100 µM and 100 µg/µL, respectively, in a Dounce homogenizer. Once samples were homogenized, they were centrifuged at 1000× *g* for 5 min and 4 °C in a Beckman JA 21 centrifuge (Coulter, Madrid, Spain). After that, the supernatant was rescued to perform another centrifugation, this time for 30 min at 27,000× *g* and 4 °C. Both fractions were collected: the supernatant corresponding to the cytosol and the pellet corresponding to the plasmatic membrane. Finally, the protein concentration of both fractions was quantified following the Lowry method, using bovine serum albumin (BSA) as a standard.

### 2.6. Western Blot Assay

Plasmatic membrane and cytosolic fractions were separated by 10% polyacrylamide gel electrophoresis (PAGE) with sodium dodecyl sulfate (SDS) as a detergent, loading 30 µg of protein from each sample. Then, the protein content in the gel was transferred to a nitrocellulose or PVDF membrane (iBlotTM Gel Transfer Stack, nitrocellulose, or iBlotTM Gel Transfer Stack PVDF, regular size) using the iBlotTM Dry Blotting System (Invitrogen, Barcelona, Spain). After blocking the membranes with 5% skimmed milk dissolved in water for 60 min at room temperature, they were washed three times for 10 min each with phosphate-buffered saline (PBS) at 1× concentration. Subsequently, the membranes were incubated overnight at 4 °C while shaking with the following primary antibodies: Anti-Adenosine A_1_ Receptor antibody (1:1000, ab124780 from Abcam, Cambridge, UK), Anti-Adenosine A_2A_ Receptor antibody (1:1000, ab79714 from Abcam), Anti-PKA antibody (1:1000, ABIN1532391 from Antibodies online, Limerick, PA, USA), β-Actin antibody (1:5000, ab8226 from Abcam), and GAPDH antibody (1:1000 from Abcam, ab8245) used as gel loading control. Once this time elapsed, three more washes with PBS were conducted before incubating with the secondary antibody for 1 h at room temperature. These antibodies were anti-rabbit (GAR) or anti-mouse (GAM) IgG, depending on the species in which the primary antibody was produced, coupled to the horseradish peroxidase enzyme (1:5000, GARPO 172-1019, and GAMPO 170-6516 from Bio-Rad). After this incubation, membranes were washed again three times with PBS, and the respective protein bands were visualized using the ECL chemiluminescent detection kit from GE Healthcare (Madrid, Spain), incubated for three minutes with the mixture of both reagents of the kit in a 1:1 ratio, and then revealed using a G:Box camera. Also, quantification of the optical density of the bands was conducted using Gene-Tools software version number 4.03.02.0 (Syngene, Bristol, UK). The results of the densitometry were expressed as percentages of control values.

### 2.7. Adenylate Cyclase Activity Assay

Adenylate cyclase activity determination in C6 and SH-SY5Y plasma membranes was performed with 20 µg of protein in a final volume of 250 µL of 50 mM Tris-HCl buffer pH 7.4, 10 mM creatine phosphate, 1 mM Dithiothreitol (DTT), 1 mg/mL BSA, 5 mM MgCl_2,_ 1 mg/mL creatine kinase, and 0.1 mM of the selective phosphodiesterase inhibitor Ro 20/1724. Previously, the plasma membrane samples were incubated with the adenosine deaminase enzyme (ADA) (0.2 U/mg protein) for 30 min at 37 °C to eliminate endogenous adenosine from the samples. Subsequently, they were incubated for 6 min at 37 °C in the absence (basal activity) or presence of 10 µM forskolin to stimulate the activity and 10 µM forskolin plus CPA at 10 µM to see the inhibition through the A_1_ receptor, which could also activate A_2_ receptors, followed by a 200 µM ATP incubation for 10 min at 37 °C. The samples were boiled at 100 °C for 3 min in order to stop the reaction. Then, centrifugation was carried out for 4 min at 12,000× *g* and 4 °C, and the supernatant (50 µL) was used for the determination of the 3′-5′ cyclic adenosine monophosphate (cAMP) accumulation. Briefly, samples were incubated with 0.25 pmol of [^3^H] cAMP and 6.25 µg of AMP-dependent protein kinase (PKA) in a final assay volume of 200 µL of determination buffer (50 mM Tris-HCl pH 7.4, 4 mM ethylenediaminetetraacetic acid (EDTA)) for 2–18 h at 4 °C. Standard samples were prepared in the same buffer in the concentration range of 0–16 pmol. The reaction was stopped by rapid filtration through Whatman GF/B filters, previously preincubated with 0.3% polyethyleneimine using a Filter Mate Harvester (PerkinElmer, Shelton, CT, USA) for at least 30 min. Finally, for the radioactivity measurement, scintillation liquid was added to a Micro Beta JET counter (PerkinElmer) using Betaplate Scint (PerkinElmer). Results of basal activity are presented as pmol cAMP/mg protein·min, forskolin-mediated activity is presented as percentage respect to basal activity, and CPA-mediated inhibition is presented as percentage respect to forskolin-stimulated activity.

### 2.8. 5′-Nucleotidase Activity Assay

Membrane or cytosolic fractions (20 µg protein) from C6 or SH-SY5Y cell lines were preincubated in a reaction medium consisting of 50 mM Tris-HCl, 5 mM MgCl_2_, pH 9, at 37 °C for 10 min with shaking. The reaction was started with the addition of 10 mM AMP (500 µM final concentration) and stopped by the addition of 10% trichloroacetic acid 20 min later. Once stopped, samples were kept on ice (at 4 °C) for at least 10 min. Later, samples were centrifuged at 12,000 rpm for 4 min at 4 °C. Finally, 40 µL of each sample supernatant was collected to quantify the inorganic phosphate (Pi) release, using KH_2_PO_4_ as the Pi standard. All experiments were performed in duplicate. 5′-nucleotidase activity is expressed as nmol Pi released/min·mg protein.

### 2.9. Statistical and Data Analysis

Statistical analyses were performed using an unpaired two-tailed Student’s *t*-test using GraphPad Prism 8. The results are expressed as the mean ± standard error of the mean (SEM). Differences between mean values were considered statistically significant at *p* < 0.05.

## 3. Results

### 3.1. Effect of Xn on C6 and SH-SY5Y Cell Lines Viability

**Figure 1:**
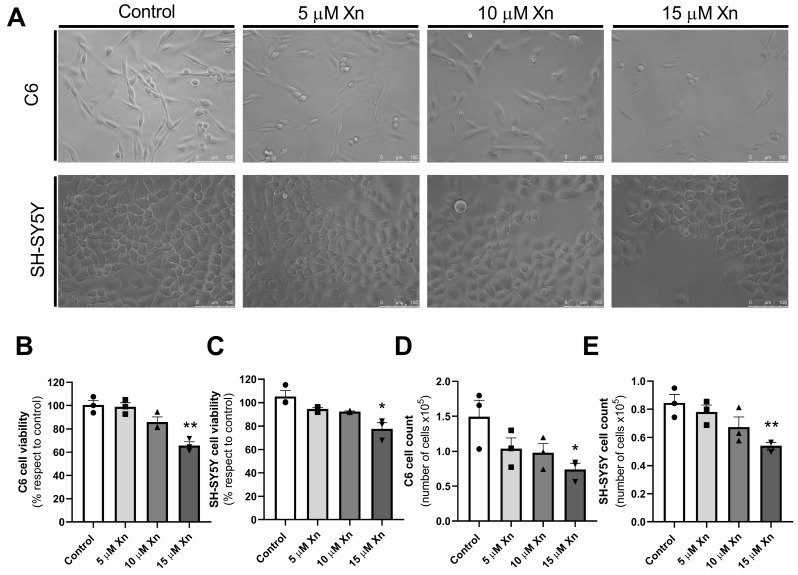
Effect of Xn on viability and cell number of C6 (panel (**A**) top row and panel (**B**) and (**D**), respectively) and SH-SY5Y (panel (**A**) bottom row and panel (**C**) and (**E**), respectively) cell lines. Cells were treated with different concentrations of Xn, and cell viability was assayed. Histograms showing data, expressed as percentages with respect to control values (**B**,**C**) or the number of cells ×10^5^ (**D**,**E**), correspond to the mean ± SEM of three different experiments carried out in sextuplicate. Individual data are shown in circles, squares and triangles. * *p* < 0.05 and ** *p* < 0.01 are significantly different from the control values according to the Student’s *t*-test.

First, a study of the cell viability of C6 and SH-SY5Y cells in the presence of different concentrations of Xn was conducted in order to establish a proper concentration that has no severe toxicity to these cell lines. Previous results of our group showed no significant effect of 10 µM Xn in these cell lines; therefore, 5 and 15 µM Xn were tested [28]. Results presented in Figure 1 show a significant decrease in cell viability when both C6 and SH-SY5Y cells were exposed to 15 µM of Xn (C6: 100.0 ± 3.9 vs. 65.8 ± 3.1%, * *p* < 0.05; SH-SY5Y: 100.0 ± 4.7 vs. 77.8 ± 5.1%, * *p* < 0.05. Panels Figure 1B and Figure 1C, respectively). However, no significant changes in viability were observed when exposing cells to 5 or 10 µM of Xn. The changes in the number of cells were consistent with the results of the viability tests, as we can see in panels Figure 1D and Figure 1E for C6 and SH-SY5Y cell lines, respectively.

### 3.2. Effect of Xn on Adenosine Receptor Gene Expression

Once viability assays were conducted and after bibliography research to choose a concentration of Xn that had reported beneficial effects in other cell lines such as PC12 (0.1–10 µM), cementoblasts (10 nM–10 µM), or HT29 (0.2–8 µM) [7,29,30], a 10 µM concentration of Xn was chosen for subsequent experiments. As adenosine receptors have been widely studied in neurodegenerative pathologies, we wanted to investigate if this flavonoid was able to modulate the expression of these receptors in C6 and SH-SY5Y cell lines. Thus, RT-qPCR assays were performed. Also, adenylate cyclase 1 enzyme isoform expression was tested.

**Figure 2:**
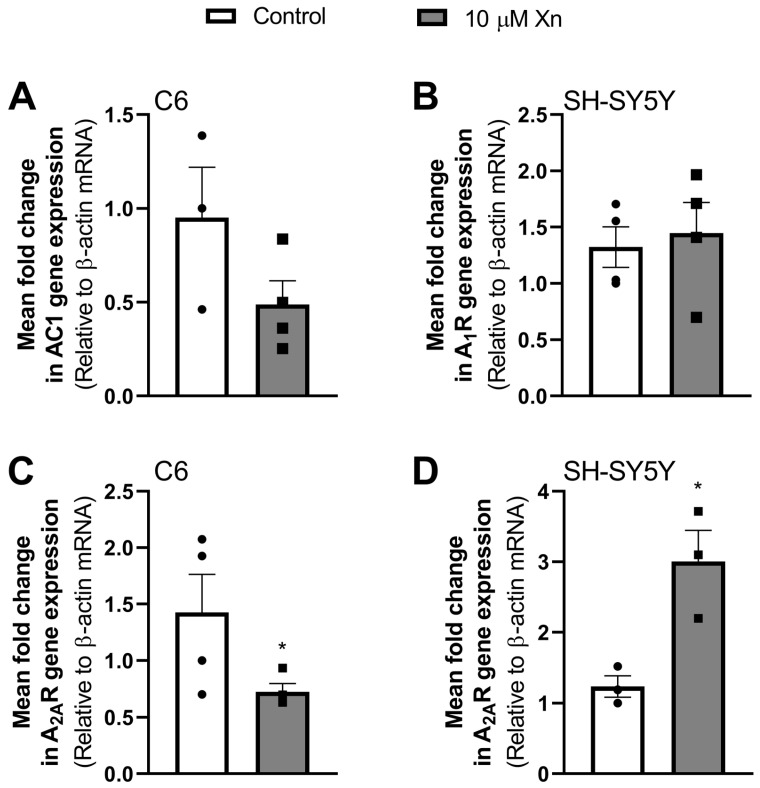
Gene expression of A_1_ (panel (**B**)) and A_2A_ (panels (**C**,**D**)) adenosine receptors and AC1 enzyme isoform (panel (**A**)) in C6 and SH-SY5Y cell lines. The expression of the β-actin gene was used as an endogenous control for each sample. Histograms show data corresponding to the mean ± SEM of three–four different experiments performed in duplicate. Individual data are shown in circles and squares. * *p* < 0.05 is significantly different according to the Student’s *t*-test.

Results presented in Figure 2 show an increase in A_2A_ receptor gene expression (1.2 ± 0.1 vs. 3.0 ± 0.4 relative units, *p* < 0.05) in SH-SY5Y (panel D), while no changes were observed in the A_1_ receptor measured in this cell line (panel B). On the other hand, A_2A_R expression in the C6 cell line was significantly reduced (0.9 ± 0.3 vs. 0.5 ± 0.1 relative units, *p* < 0.05) by Xn treatment (panel C), whereas AC1 was not significantly modulated (0.95 ± 0.27 vs. 0.49 ± 0.13 relative units, *p* = 0.1711, panel A). However, under our experimental conditions, we were unable to amplify the A_1_R and AC1 genes for C6 and SH-SY5Y cells, respectively.

### 3.3. Effect of Xn on Adenosine Receptors Levels in Plasma Membrane

**Figure 3:**
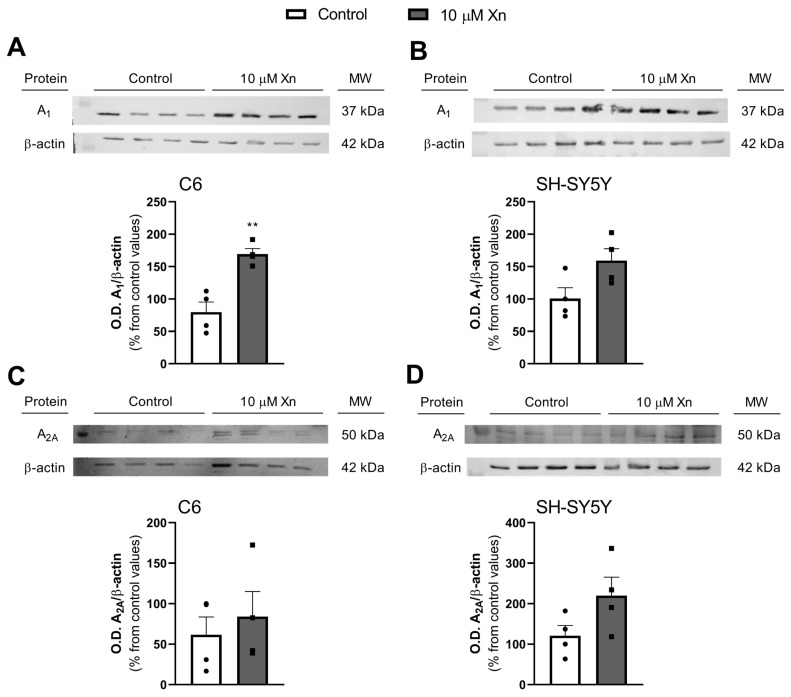
Adenosine A_1_ and A_2A_ receptor levels in plasma membranes from C6 and SH-SY5Y after Xn exposure. Western blot analysis was performed with plasma membranes obtained after Xn treatment for 24 h for A_1_R (panel (**A**) and (**B**) for C6 and SH-SY5Y cell lines, respectively) and A_2A_R (panel (**C**,**D**) for C6 and SH-SY5Y cell lines, respectively). Graphs representing data, expressed as % from control values, correspond to the mean ± SEM of four experiments carried out with different plasmatic membrane isolations normalized using β-actin as the control loading. Individual data are shown in circles and squares.** *p* < 0.01 is significantly different from the control according to the Student’s *t*-test.

The effect of Xn on adenosine receptor levels in plasma membranes from both cell lines was analyzed by Western blotting. As shown in Figure 3 (panel A), adenosine A_1_ receptor levels were significantly increased in the C6 cell line (79.7 ± 15.7 vs. 169.3 ± 8.4% from control values, *p* < 0.01), while in SH-SY5Y (panel B), it was also observed a tendency to increase, although not significant (100.8 ± 16.5 vs. 159.3 ± 18.3% from control values, *p* = 0.06). On the other hand, adenosine A_2A_ receptor levels were not significantly affected by the 10 µM Xn treatment, neither in C6 (61.58 ± 22.00 vs. 84.04 ± 31.08% from control values, *p* = 0.5768) nor in SH-SY5Y (120.73 ± 25.41 vs. 219.83 ± 45.58% from control values, *p* = 0.1063) cell lines (panels Figure 3C,D, respectively), although a tendency to increase has been shown for SH-SY5Y cells.

### 3.4. Effect of Xn on Adenylate Cyclase Activity and PKA Levels

**Figure 4:**
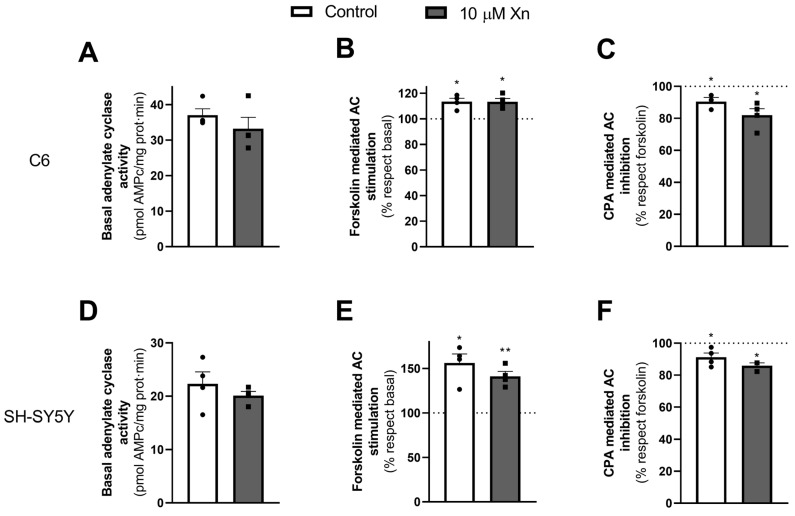
Adenylate cyclase activity in plasmatic membrane fractions from C6 and SH-SY5Y cells. Adenylate activity was analyzed in plasma membranes after Xn treatment for 24 h. Histograms show data representing basal (panels (**A**,**D**)), forskolin-stimulated (panels (**B**–**E**)), and CPA inhibited over forskolin-stimulated activity (panels (**C**,**F**)) in C6 and SH-SY5Y cell lines, respectively. Data are expressed as pmol cAMP/mg protein·min for basal activity, as a percentage of basal values for forskolin-stimulated, and as % from forskolin-stimulated for CPA-inhibited activity, and they correspond to the mean ± SEM of three–four experiments performed using different plasma membrane isolations. Individual data are shown in circles and squares. Dashed line in B, E correspond to basal value, set to 100 %; dashed line in C, F correspond to forskolin-stimulated value, set to 100 % * *p* < 0.05 and ** *p* < 0.01 are significantly different from basal (panels (**B**,**E**)) or forskolin-stimulated activity (panels (**C**,**F**)) according to the Student’s *t*-test.

Next, adenylate cyclase activity was measured to determine whether the increase in adenosine A_1_ receptor level could also affect its signaling pathway. The results are presented in Figure 4. Interestingly, no effect due to Xn treatment was observed in C6 respecting basal activity (panel Figure 4A) or forskolin-stimulated activity (panel Figure 4B). The functionality through the A_1_ receptor after stimulating with forskolin and inhibiting with the A_1_ receptor agonist, CPA, also remained unaltered (panel Figure 4C). The same results were obtained for the human neuroblastoma SH-SY5Y cell line (panels Figure 4D, Figure 4E, and Figure 4F, respectively).

**Figure 5:**
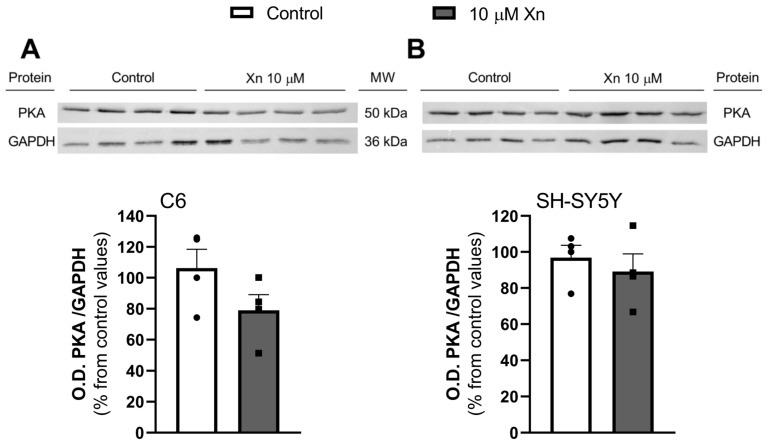
PKA levels in C6 (panel (**A**)) and SH-SY5Y (panel (**B**)) cells. Western blots were performed in cytosol fractions from cells after 10 µM Xn treatment for 24 h. Histograms show that results, expressed as % of control values, correspond to the mean ± SEM of four experiments performed using different cytosol fractions and normalized using GAPDH as the control loading. Individual data are shown in circles and squares. No differences were found according to the statistical analysis.

Moreover, protein kinase A (PKA) enzyme levels, which are involved in the downstream adenosine pathway through adenylate cyclase, were measured by Western blot. Regarding this enzyme, no changes were observed in any cell lines, as can be observed in Figure 5.

### 3.5. Effect of Xn in 5′-Nucleotidase Activity (CD73)

**Figure 6:**
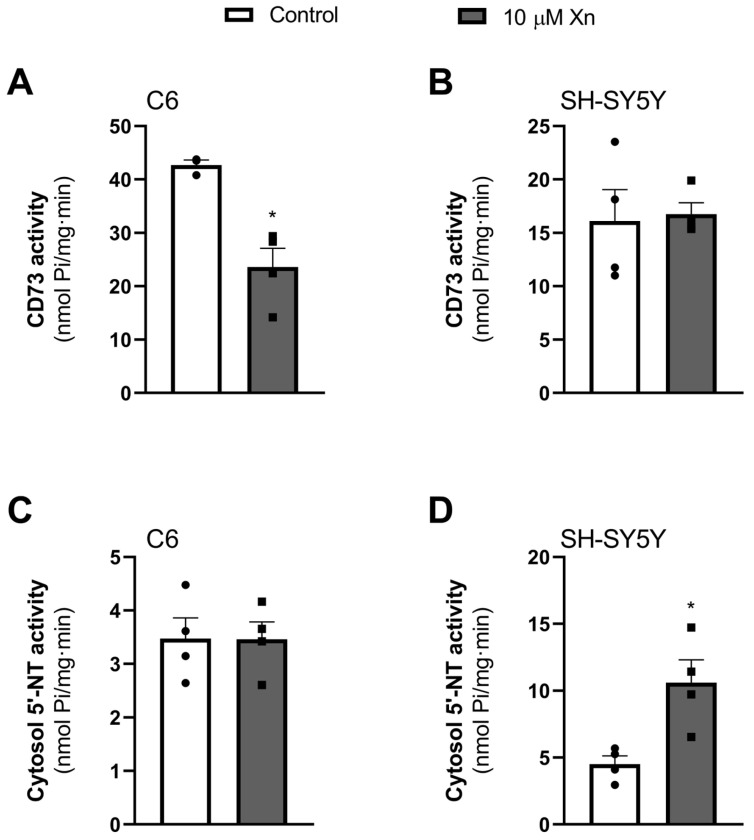
5′-Nucleotidase enzymatic activity in the plasmatic membrane (CD73) and cytosol fractions from C6 (panels (**A**,**C**)) and SH-SY5Y (panels (**B**,**D**)) cell lines, respectively, after 10 µM Xn exposure for 24 h. Results are expressed as the mean ± SEM of four different experiments performed in duplicate. Individual data are showin in circles and squares. * *p* < 0.05 is significantly different according to the Student’s *t*-test.

After assessing adenosine receptors and functionality, other enzymatic activity related to this nucleoside was studied, such as 5′-nucleotidase activity. This enzyme is responsible for degrading mainly AMP into adenosine, and it can be found attached to the plasma membrane (ecto-5′-nucleotidase, or CD73) or in the cytosolic fraction. Xn exposure at 10 µM for 24 h caused a significant decrease in CD73 activity (42.7 ± 1.15 vs. 23.6 ± 4.0 nmol Pi/mg protein·min, *p* < 0.05), while the cytosolic activity of the enzyme remains unaltered by the treatment in C6 glioma cells, as can be observed in Figure 6A and Figure 6C, respectively. As for SH-SY5Y cells, no change was observed in CD73 activity, while an increase in cytosolic activity was noted (4.5 ± 0.7 vs. 10.6 ± 1.9 nmol Pi/mg protein·min, *p* < 0.05), as shown in Figure 6B and Figure 6D, respectively.

## 4. Discussion

Beer is one of the most consumed beverages worldwide, and 80% of its characteristic bitterness comes from the addition of hops during the brewing process [31]. Its polyphenolic compounds content ranges from 74 to 256 mg/L, derived from hops (30%) and malt (70–80%) [32], and Xn is its major prenylated flavonoid, being beer the main source of this molecule [33].

Despite the high prevalence of Alzheimer’s disease in the human population, research conducted to date has yet to discover an effective drug capable of halting the progression of the disease. Consequently, efforts have been made to develop new cellular models that could serve as potential predictive platforms for the development of novel drugs. As a result, various cell lines with neuronal properties have been extensively utilized to replicate the pathological processes of Alzheimer’s disease. Among these cell lines are PC12, as well as those utilized in this study, SH-SY5Y and C6, derived from rat glioma [34]. However, a more precise study of its action over viability in these cell lines was needed to prove if this molecule by itself could have a potential positive impact on neurodegeneration without affecting viability. It has shown protective effects against the toxicity of several molecules, such as methylglyoxal in osteoblastic cells, at which Xn by itself at 1 µM caused a significant decrease in this cell line viability [35], or in cementoblasts, which started to decrease their viability at 4 µM Xn [29]. It also caused a significant diminution of viability in PC12 neurons like rat pheochromocytoma cells at concentrations greater than 2 µM [7]. Thus, in our case, both cell lines performed equally well in terms of viability when adding Xn for a 24 h treatment. Therefore, a 10 µM concentration was employed for the subsequent experiments in C6 and SH-SY5Y cell lines in order to avoid this cytotoxicity and to ensure the usage of a concentration of this flavonoid, which has reported effects in other cell lines.

Adenosine is a neuromodulator nucleoside that acts through four different receptors, with A_1_R and A_2A_R being the main ones responsible for the adenosine function in the brain [36]. A_1_R has been known for its neuroprotective effect [37], acting pre-synaptically, decreasing the calcium influx, and, in turn, glutamate release, among other functions [38]. In fact, some studies carried out in the post-mortem brains of AD patients have observed that adenosine A_1_ receptors are decreased, even though their expression remains unaltered [39], promoting an environment of excess glutamate release that ends up in an excitotoxicity process [13]. Other works propose that simultaneously boosting A_1_R activity and blocking A_2A_R may accomplish maximal neuroprotection, preventing brain damage [15]. In fact, other polyphenolic bioactive compounds such as resveratrol have shown these effects in in vitro [40] and in vivo studies [41], and caffeine has also been proposed as neuroprotective mainly due to its A_2A_R antagonism [18,42].

Regarding the presence of adenosine receptors in the plasma membrane, A_1_ levels were significantly increased, while no changes in A_2A_ levels were found. Thus, an increase in A_1_R membrane levels could indicate a possible neuroprotective effect of Xn. However, different results were found between cell lines in adenosine receptor gene expression. Our data show a clear modulation of A_2A_ expression by Xn, while no modulation in A_1_ gene expression in the SH-SY5Y cell line was observed. As reported, these results are not paralleled by changes in the corresponding presence of these receptors in the plasma membrane. The discrepancies between gene expression and their respective protein levels have been previously reported in different cell lines, such as HeLa [40], and tissues by our group [39,41,43]. A possible explanation for these results could be that Xn exposure time is not enough to observe an effect on the translation of the A_2A_ receptor. Also, for the A_1_ receptor, the stability of its mRNA could have been modulated by the Xn treatment. Despite this, the different effects of this flavonoid on A_2A_ expression should be considered since it acts differently depending on the cell type or perhaps the host species of these cells.

Once we studied the modulation of receptor expression and their membrane levels, AC activity at different conditions was measured to probe if Xn was affecting the functionality of adenosine receptors through this pathway. AC activity is crucial for the Ca^2+^ and cAMP second messenger balance inside the cell [44], and dysregulation of this activity or PKA activity may translate into structural and functional abnormalities [45]. Therefore, their modulation has been considered a promising target for drugs with neuroregenerative activity for the treatment of dementia [46]. Also, some studies remark that AC activity is diminished in neurodegenerative diseases in some brain areas [47]; hence, a possible modulation of this activity by Xn must be studied. Interestingly, no effect was observed in adenylate cyclase basal activity after Xn treatment nor in its activity through the A_1_ adenosine receptor in any of the cell lines tested under our conditions. Additionally, PKA levels were not affected.

Regarding 5′-nucleotidase activity, a different modulation occurred when treating cells for 24 h with Xn in C6 or SH-SY5Y cell lines, decreasing the activity of the ecto-5′-nucleotidase or CD73 in the rat model, the major enzyme responsible for the extracellular AMP degradation into adenosine [48], while increasing its activity in the cytosolic fraction of the SH-SY5Y human cellular model. The role of ecto-5′-nucleotidases in pathologies has been widely described as they participate in a large variety of processes, such as extracellular availability of nucleosides [49], synaptogenesis [50], and cell proliferation [51], and it has been described as decreased in some early stages of AD [24]. According to that, this enzyme activity has been targeted in several neurodegenerative diseases, as A_2A_R is mainly activated by adenosine coming from CD73 ecto-5′-nucleotidase activity [25,52]. Even a physical interaction through coimmunoprecipitation between this enzyme and the receptor has been described [53]. It has been described how the adenosine produced by the catabolism of ATP directly activates the A_2A_ receptor, causing synaptic disruption and memory dysfunction in a mouse model of AD [23], proposing CD73 activity as a novel target for the modulation of A2AR function. Other works reported the importance of this ecto-5′-nucleotidase in the regulation of A_2A_ function in animal models of Parkinson’s disease [25], brain damage produced by convulsive activity, which leads to an extracellular ATP increase and, therefore, affects the CD73-A_2A_R pathway [54], and even the damage caused by repeated stress on adult rats [55]. In addition, this enzyme is important in embryonic brain development, taking part in the migration of cortical projection neurons [56]. These studies comment on the role of this enzyme in multiple neurological processes and propose it as a potential target for several diseases. Thus, a decrease in this activity could moderate A_2A_ function and become another crucial factor contributing to the potential neuroprotective effect of Xn. Concerning the decrease in cytosolic 5′-nucleotidase activity observed in SH-SY5Y cells following Xn exposure, this effect may also contribute to a protective effect against Alzheimer’s disease since a previous study conducted by our group has also revealed a significant reduction in cytosolic 5′-nucleotidase activity in the frontal cortex of human patients with Alzheimer’s [24].

In addition, due to the antiproliferative and antioxidant effects provided by Xn in different cell lines, it could also be a promising antitumoral molecule since gliomas are the most frequent malignant tumors of the CNS in adults, and currently, there are no strategies to achieve their successful eradication [57].

## 5. Conclusions

In summary, here we describe a novel route in which Xn could act in preventing neurodegeneration as well as its reported prevention of oxidative stress, both of which are common signs of dementia, proposing this molecule as a potential neuroprotective agent through the adenosine pathway modulation in different cell lines.

## Data Availability

The data presented in this study are available on request from the corresponding author due to privacy.
